# Data on the catalytic mechanism of thiol peroxidase mimics

**DOI:** 10.1016/j.dib.2016.05.037

**Published:** 2016-05-24

**Authors:** B. Zadehvakili, N.M. Giles, J.P. Fawcett, G.I. Giles

**Affiliations:** a School of Pharmacy, University of Otago, Dunedin, New Zealand; b Department of Pharmacology and Toxicology, Otago School of Medical Sciences, University of Otago, Dunedin, New Zealand

## Abstract

We have recently reported SAR data describing the pharmacological activity of a series of phenyl alkyl selenides and tellurides which catalyse the oxidation of thiols by hydrogen peroxide (H_2_O_2_), “The design of redox active thiol peroxidase mimics: dihydrolipoic acid recognition correlates with cytotoxicity and prooxidant action” B. Zadehvakili, S.M. McNeill, J.P. Fawcett, G.I. Giles (2016) [1]. This thiol peroxidase (TPx) activity is potentially useful for a number of therapeutic applications, as it can alter the outcome of oxidative stress related pathologies and modify redox signalling. This article presents data describing the molecular changes that occur to a TPx mimic upon exposure to H_2_O_2_, and then the thiol mercaptoethanol, as characterised by UV–vis spectroscopy and HPLC retention time.

**Specifications Table**

**Table:** 

Subject area	*Pharmacology.*
More specific subject area	*Redox drugs.*
Type of data	*Figures.*
How data was acquired	*Jenway 6715 UV–vis spectrometer. Shimadzu LC-10AT HPLC with SPD-M10A diode array detector and Gemini C18 column (5 µm, 100 Å, 100 × 4.6 mm).*
Data format	*Raw.*
Experimental factors	*Drug solutions treated with H*_*2*_*O*_*2*_*and 2-mercaptoethanol.*
Experimental features	*Changes to TPx mimic UV–vis spectra and HPLC retention time following oxidation and reduction. Background corrected UV–vis absorbance spectra, raw HPLC traces acquired at 270 nm after setting the channel current to zero.*
Data source location	*University of Otago, Dunedin, New Zealand.*
Data accessibility	*Data provided in article.*

**Value of the data**

• TPx mimics display antioxidant, pro-oxidant and cytotoxic properties and are being developed as therapeutic agents. The data describe methodology to spectroscopically characterize their reactions with H_2_O_2_ and thiol substrates, which will be useful for future investigations into the chemical mechanism of this drug class.
• Structure-Activity Relationship (SAR) studies are currently being developed to explore the pharmacological activity of TPx mimics. The TPx mimic catalytic cycle consists of an initial oxidation step by H_2_O_2_, followed by reduction by a thiol to regenerate the starting mimic. The data provide information quantifying variations in TPx mimic hydrophobicity during this cycle. This parameter has never before been applied to SAR studies, and has the potential to improve our understanding of drug pharmacology.
• The data provide information on the catalytic cycle and biophysical properties of phenyl butyl telluride, a TPx mimic currently being evaluated as a prooxidant drug. This is of wide-ranging interest to researchers investigating the application of therapeutic agents to manipulate the cellular redox state.

## 1 Data

**Fig. 1:**
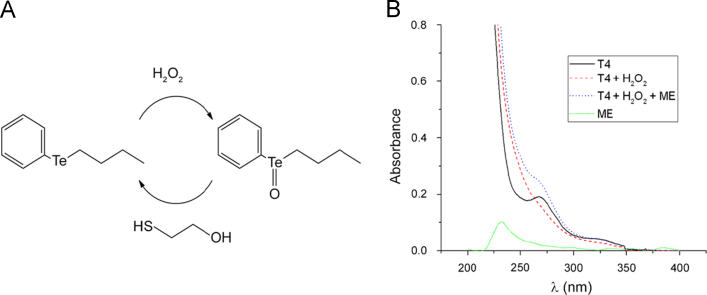
Reversibility of the TPx catalytic cycle. **A**: Catalytic cycle of TPx mimic T4. The reaction takes place in two steps, initially the T4 telluride is oxidised to a telluroxide by H_2_O_2_[5]. The metal centre is then regenerated by reduction with a thiol [5], in this case mercaptoethanol (ME). **B**: Spectroscopic features characteristic of the TPx redox cycle. T4 (50 µM, black trace) reacted with H_2_O_2_ (1 mM) to form an oxidised intermediate (red trace). The TPx mimic was then regenerated by the addition of ME (1 mM, blue trace). The spectrum of ME alone (green trace) is shown for comparison.

**Fig. 2:**
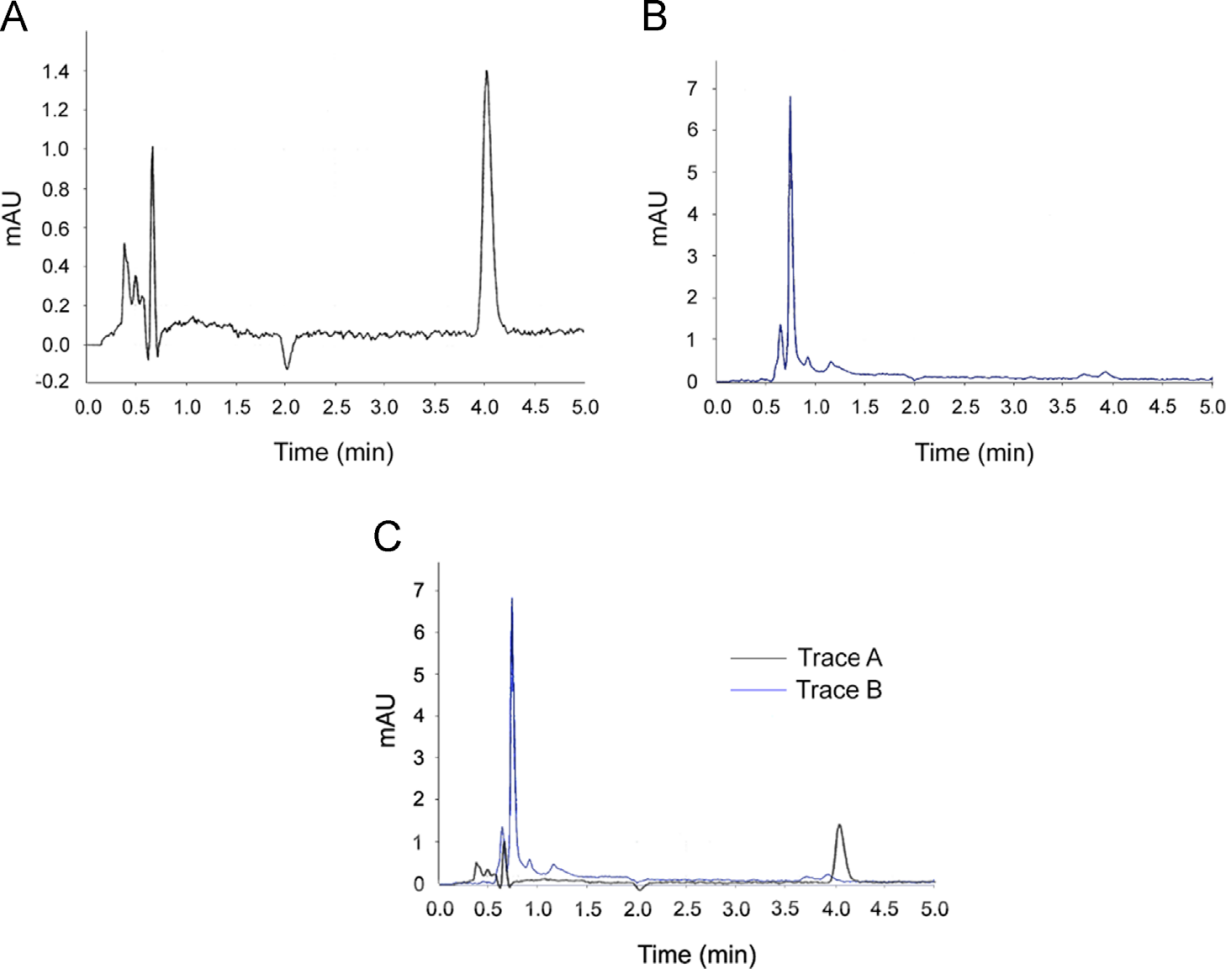
Effect of oxidation on T4 hydrophobicity. **A**: Initial chromatogram of T4, **B**: chromatogram of T4 after the addition of H_2_O_2_ (1 mM) and incubation for 5 min, **C**: Superimposition of **A** (initial chromatogram) and **B** (chromatogram following addition of H_2_O_2_), the axis of **A** re-scaled for alignment.

We present UV–vis spectra which characterize the initial, oxidised and regenerated forms of phenyl butyl telluride (T4). This is accompanied by HPLC data revealing a change in molecular hydrophobicity as T4 is oxidised. See Fig. 1, Fig. 2.

### 1.1 Experimental design, materials and methods

#### 1.1.1 Synthesis of phenyl butyl telluride (T4)

T4 was prepared according to an established procedure [2] and compound structure confirmed by comparison to published data [3].

#### 1.1.2 Standardisation of H_2_O_2_ solutions

The concentration of a commercial H_2_O_2_ solution (Sigma–Aldrich, Auckland, New Zealand) was determined by UV–vis absorbance (*ε*_240_ = 43.6 M^−1^cm^−1^ [4]).

#### 1.1.3 UV–vis Spectroscopy

T4 was dissolved in methanol and then diluted to 50 µM in methanol. UV–vis spectra were acquired over the wavelength range 200–400 nm with a scan rate of 4 nm/s.

#### 1.1.4 HPLC Analysis

T4 was initially dissolved and diluted in acetonitrile to a concentration of 100 µM. A 20 µl aliquot of this solution was then injected into an HPLC system under isocratic conditions (75:25 v/v acetonitrile:water) with a flow rate of 2.5 ml/min. Compound elution was monitored over 5 min at 270 nm.
